# Supplementary material for: Phylogenetic and Morphological Analysis of Wing Base Articulation in Vespidae (Hymenoptera): A Cladistic Approach
Source: Insects. 2025 Dec 27;17(1):39. doi: 10.3390/insects17010039 (PMC12842110; doi:10.3390/insects17010039)
Supplement: Supplementary file 1 [file insects-17-00039-s001.zip › Characters Matrix.docx]

**S1. Data matrix for phylogenetic analysis**

| Species | 1 | 2 | 3 | 4 | 5 | 6 | 7 | 8 | 9 | 10 | 11 | 12 | 13 | 14 | 15 | 16 | 17 | 18 | 19 |
| --- | --- | --- | --- | --- | --- | --- | --- | --- | --- | --- | --- | --- | --- | --- | --- | --- | --- | --- | --- |
| *Vespula germanica* | 3 | 0 | 0 | 1 | 2 | 1 | 1 | 1 | 2 | 0 | 0 | 0 | 1 | 0 | 1 | 0 | 1 | 0 | 0 |
| *Provespa barthelemyi* | 3 | 0 | 0 | 0 | 2 | 1 | 1 | 1 | 2 | 0 | 0 | 1 | 1 | 0 | 1 | 0 | 1 | 1 | 0 |
| *Vespa analis* | 3 | 0 | 0 | 0 | 2 | 1 | 1 | 1 | 2 | 0 | 0 | 1 | 1 | 0 | 1 | 0 | 1 | 1 | 0 |
| *Vespa velutina* | 3 | 0 | 0 | 0 | 2 | 1 | 1 | 1 | 2 | 0 | 0 | 1 | 1 | 0 | 1 | 0 | 1 | 1 | 0 |
| *Vespa ducalis* | 3 | 0 | 0 | 0 | 2 | 1 | 1 | 1 | 2 | 0 | 0 | 1 | 1 | 0 | 1 | 0 | 1 | 1 | 0 |
| *Vespa mandarinia* | 3 | 0 | 0 | 0 | 2 | 1 | 1 | 1 | 2 | 0 | 0 | 1 | 1 | 0 | 1 | 1 | 1 | 0 | 0 |
| *Vespa basalis Smith* | 3 | 0 | 0 | 0 | 2 | 1 | 1 | 1 | 2 | 0 | 0 | 1 | 1 | 0 | 1 | 1 | 1 | 0 | 0 |
| *Polistes jokahamae* | 1 | 0 | 0 | 2 | 1 | 1 | 1 | 1 | 0 | 0 | 0 | 1 | 2 | 0 | 1 | 0 | 0 | 1 | 1 |
| *Polistes rothneyi* | 1 | 0 | 0 | 2 | 1 | 1 | 1 | 1 | 0 | 0 | 0 | 0 | 2 | 0 | 1 | 0 | 1 | 1 | 1 |
| *Provespa barthelemyi* | 3 | 0 | 0 | 3 | 1 | 1 | 1 | 1 | 0 | 0 | 0 | 1 | 0 | 1 | 0 | 1 | 1 | 1 | 0 |
| *Polistes megei* | 1 | 0 | 0 | 2 | 1 | 1 | 1 | 1 | 0 | 0 | 0 | 1 | 2 | 0 | 1 | 0 | 1 | 1 | 1 |
| *Polistes tenuispunctia* | 1 | 0 | 0 | 2 | 1 | 1 | 1 | 1 | 0 | 0 | 0 | 1 | 2 | 0 | 1 | 0 | 1 | 1 | 1 |
| *Polistes snelleni* | 1 | 0 | 0 | 2 | 1 | 1 | 1 | 1 | 0 | 0 | 0 | 1 | 2 | 0 | 1 | 0 | 1 | 1 | 1 |
| *Stenogater* | 2 | 1 | 1 | 2 | 0 | 0 | 0 | 0 | 1 | 0 | 0 | 0 | 0 | 1 | 0 | 1 | 0 | 1 | 1 |
| *Oreumenes decoratus* | 0 | 1 | 1 | 2 | 0 | 0 | 0 | 0 | 0 | 1 | 1 | 0 | 0 | 0 | 0 | 1 | 0 | 1 | 1 |
| *Dolicovespula*  *flora* | 3 | 0 | 0 | 0 | 2 | 1 | 1 | 1 | 2 | 0 | 0 | 1 | 1 | 0 | 1 | 0 | 1 | 1 | 0 |
| *Xyela Sp.* | 4 | 1 | 0 | 0 | 2 | 0 | 0 | 0 | 1 | 0 | 0 | 0 | 0 | 1 | 1 | 1 | 1 | 3 | 1 |

| Species | 20 | 21 | 22 | 23 | 24 | 25 | 26 | 27 | 28 | 29 | 30 | 31 | 32 | 33 | 34 | 35 | 36 | 37 | 38 |
| --- | --- | --- | --- | --- | --- | --- | --- | --- | --- | --- | --- | --- | --- | --- | --- | --- | --- | --- | --- |
| *Vespula germanica* | 0 | 1 | 2 | 1 | 1 | 0 | 0 | 0 | 1 | 0 | 0 | 1 | 0 | 0 | 0 | 1 | 0 | 0 | 0 |
| *Provespa barthelemyi* | 0 | 1 | 2 | 0 | 0 | 0 | 0 | 0 | 1 | 0 | 0 | 1 | 0 | 0 | 0 | 1 | 0 | 0 | 0 |
| *Vespa analis* | 0 | 1 | 2 | 0 | 0 | 0 | 0 | 0 | 1 | 0 | 1 | 1 | 0 | 1 | 0 | 1 | 0 | 0 | 0 |
| *Vespa velutina* | 0 | 1 | 2 | 0 | 0 | 0 | 0 | 1 | 1 | 0 | 1 | 1 | 0 | 0 | 0 | 1 | 0 | 0 | 0 |
| *Vespa ducalis* | 0 | 1 | 2 | 0 | 0 | 0 | 0 | 0 | 1 | 0 | 1 | 1 | 0 | 1 | 0 | 1 | 0 | 0 | 0 |
| *Vespa mandarinia* | 0 | 1 | 2 | 1 | 0 | 0 | 0 | 0 | 1 | 0 | 1 | 1 | 0 | 1 | 0 | 1 | 0 | 0 | 0 |
| *Vespa basalis Smith* | 0 | 1 | 2 | 0 | 0 | 0 | 0 | 0 | 1 | 0 | 1 | 1 | 0 | 1 | 0 | 1 | 0 | 0 | 0 |
| *Polistes jokahamae* | 0 | 1 | 0 | 0 | 0 | 1 | 0 | 0 | 0 | 0 | 1 | 0 | 1 | 1 | 1 | 1 | 0 | 1 | 1 |
| *Polistes rothneyi* | 0 | 1 | 0 | 0 | 0 | 1 | 0 | 1 | 0 | 0 | 0 | 0 | 1 | 1 | 1 | 1 | 0 | 1 | 1 |
| *Provespa barthelemyi* | 0 | 1 | 0 | 0 | 0 | 1 | 0 | 0 | 0 | 0 | 1 | 0 | 1 | 1 | 1 | 1 | 0 | 1 | 1 |
| *Polistes megei* | 0 | 1 | 0 | 0 | 0 | 1 | 0 | 0 | 0 | 0 | 1 | 0 | 1 | 1 | 1 | 1 | 0 | 1 | 1 |
| *Polistes tenuispunctia* | 0 | 1 | 0 | 1 | 1 | 1 | 0 | 0 | 0 | 0 | 1 | 0 | 1 | 1 | 1 | 1 | 0 | 1 | 1 |
| *Polistes snelleni* | 0 | 1 | 0 | 0 | 0 | 1 | 0 | 0 | 0 | 0 | 1 | 0 | 1 | 1 | 1 | 1 | 0 | 1 | 1 |
| *Stenogater* | 0 | 2 | 1 | 1 | 1 | 0 | 1 | 0 | 0 | 1 | 0 | 1 | 1 | 0 | 1 | 0 | 1 | 1 | 1 |
| *Oreumenes decoratus* | 1 | 0 | 1 | 1 | 1 | 0 | 1 | 0 | 1 | 1 | 0 | 1 | 1 | 0 | 1 | 0 | 1 | 1 | 1 |
| *Dolicovespula*  *flora* | 0 | 1 | 2 | 0 | 0 | 0 | 0 | 0 | 1 | 0 | 0 | 0 | 1 | 0 | 0 | 0 | 1 | 0 | 0 |
| *Xyela Sp.* | 1 | 0 | 2 | 1 | 3 | 0 | 1 | 1 | 0 | 4 | 2 | 1 | 0 | 0 | 0 | 4 | 1 | 1 | 0 |

|  |  |  |  |  |  |  |
| --- | --- | --- | --- | --- | --- | --- |

| Species | 39 | 40 | 41 | 42 |  |  |  |  |  |  |  |  |  |  |  |  |  |  |  |
| --- | --- | --- | --- | --- | --- | --- | --- | --- | --- | --- | --- | --- | --- | --- | --- | --- | --- | --- | --- |
| *Vespula germanica* | 1 | 0 | 0 | 0 |  |  |  |  |  |  |  |  |  |  |  |  |  |  |  |
| *Provespa barthelemyi* | 1 | 0 | 0 | 0 |  |  |  |  |  |  |  |  |  |  |  |  |  |  |  |
| *Vespa analis* | 1 | 0 | 0 | 0 |  |  |  |  |  |  |  |  |  |  |  |  |  |  |  |
| *Vespa velutina* | 1 | 0 | 0 | 0 |  |  |  |  |  |  |  |  |  |  |  |  |  |  |  |
| *Vespa ducalis* | 1 | 0 | 0 | 0 |  |  |  |  |  |  |  |  |  |  |  |  |  |  |  |
| *Vespa mandarinia* | 1 | 0 | 0 | 0 |  |  |  |  |  |  |  |  |  |  |  |  |  |  |  |
| *Vespa basalis Smith* | 1 | 0 | 0 | 0 |  |  |  |  |  |  |  |  |  |  |  |  |  |  |  |
| *Polistes jokahamae* | 0 | 1 | 1 | 1 |  |  |  |  |  |  |  |  |  |  |  |  |  |  |  |
| *Polistes rothneyi* | 0 | 1 | 1 | 1 |  |  |  |  |  |  |  |  |  |  |  |  |  |  |  |
| *Provespa barthelemyi* | 0 | 1 | 1 | 1 |  |  |  |  |  |  |  |  |  |  |  |  |  |  |  |
| *Polistes megei* | 0 | 1 | 1 | 1 |  |  |  |  |  |  |  |  |  |  |  |  |  |  |  |
| *Polistes tenuispunctia* | 0 | 1 | 1 | 1 |  |  |  |  |  |  |  |  |  |  |  |  |  |  |  |
| *Polistes snelleni* | 0 | 1 | 1 | 1 |  |  |  |  |  |  |  |  |  |  |  |  |  |  |  |
| *Stenogater* | 1 | 1 | 1 | 1 |  |  |  |  |  |  |  |  |  |  |  |  |  |  |  |
| *Oreumenes decoratus* | 1 | 1 | 1 | 1 |  |  |  |  |  |  |  |  |  |  |  |  |  |  |  |
| *Dolicovespula*  *flora* | 0 | 0 | 1 | 0 |  |  |  |  |  |  |  |  |  |  |  |  |  |  |  |
| *Xyela Sp.* | 2 | 1 | 0 | 2 |  |  |  |  |  |  |  |  |  |  |  |  |  |  |  |

|  |  |  |  |  |  | 0 | 0 | 2 |
| --- | --- | --- | --- | --- | --- | --- | --- | --- |
